# A correlation study of beat-to-beat R-R intervals and pulse arrival time under natural state and cold stimulation

**DOI:** 10.1038/s41598-021-90056-2

**Published:** 2021-05-27

**Authors:** Rong-Chao Peng, Yi Li, Wen-Rong Yan

**Affiliations:** 1 School of Biomedical Engineering, Guangdong Medical University, Dongguan, 523808 Guangdong China; 2 School of Biomedical Sciences, The Chinese University of Hong Kong, Hong Kong, 999077 China; 3 Department of Materials Science and Engineering, City University of Hong Kong, Hong Kong, 999077 China; 4 Shenzhen Institutes of Advanced Technology, Chinese Academy of Sciences, Shenzhen, 518055 China

**Keywords:** Data processing, Data acquisition

## Abstract

Beat-to-beat R-R intervals (RRI) and pulse arrival time (PAT) provide pivotal information to evaluate cardiac autonomic functions for predicting arrhythmias and cardiovascular morbidity. However, their relationship has not been clearly understood. In this study, we simultaneously recorded electrocardiograms and photoplethysmograms on 34 subjects in the natural state, and on 55 subjects under the cold stimulation. The RRI and the PAT were calculated and then analyzed using Pearson correlation coefficient. The results showed that the RRI and the PAT were strongly correlated (*r* = 0.562) and the RRI series were 2.18 ± 0.40 beats advanced to the PAT series. After smoothing, the RRI and the PAT were more correlated in the low frequency than in the high frequency. Furthermore, when involving RRI with the phase effect, the proposed PAT based model showed better performance for blood pressure estimation. We think these results are helpful to understand the underlying regulatory mechanisms of the two cardiovascular factors, and would provide useful suggestions for non-invasive cuffless blood pressure estimation.

## Introduction

R-R intervals (R-wave peak to R-wave peak in electrocardiograms, RRI) represent the measurements of the sinus heart period in chronological or heartbeat order^1^. RRI variations between heart beats, reflect both vagal and sympathetic modulation of the heart sinus node^2^ and are commonly used to perform heart rate variability (HRV) analysis, which is a non-invasive tool for assessing the autonomic function.

Pulse transit time (PTT) is the time that the pulse travels from one site of the body to another site, and pulse wave velocity (PWV) is the propagation speed of the aortic pulse wave, i.e., the distance that the pulse travels divided by the PTT. Practically, PTT is often calculated as the time interval from a characteristic point of electrocardiogram (ECG) to a characteristic point of the peripheral pulse (usually photoplethysmogram, PPG), which is also called pulse arrival time (PAT), due to its ease of use. The PAT or PWV is an important tool to evaluate the arterial stiffness and atherosclerosis because the pulse wave travels faster when the arteries become stiffer^3^. It is also a pivotal marker of cardiovascular events in hypertension^4^ and in end-stage renal failure^5^, a good predictor for measuring long-term changes in arterial stiffness^6^, and an association factor for prediction of blood pressure (BP)^7–9^.

PAT (or PWV) and RRI (or heart rate) are both important prediction factors of cardiovascular diseases^10^, it is thus worth probing their fluctuations and investigating the relationships between their changes. Nakao et al*.* showed that PWV was positively correlated with heart rate using a univariate analysis^11^. Albaladejo et al*.* reported that a trend existed for an increase of PWV with heart rate in subjects with cardiac pace maker^12^. Lantelme et al*.* revealed that heart rate was an important factor in the intra-individual variation of PWV in elderly subjects^13^. Tang et al*.* showed PAT and RRI had a weak relation and the phase shift in PAT and RRI was 0.54 ± 0.51 cardiac beats in adult New Zealand white rabbits^14^. Drinnan et al*.* found that there was a strong correlation between beat-to-beat PAT and RRI changes when RRI was delayed with respect to PAT during paced respiration^15^. To sum up, these studies showed that RRI was of particular importance for PAT measurement in different physiological and psychological conditions.

However, the detailed relationship of RRI and PAT has not been clearly demonstrated. Little is known about their quantitative relationship in natural state and their corresponding changes when exposed to external stimuli. Therefore the aim of this study is to quantitatively investigate the relationship between the fluctuations of beat-to-beat RRI and PAT in natural state, and to compare the results with those during cold stimulation.

## Methods

### Experiments

Two experiments were performed to collect physiological signals in natural state and under cold stimulation. The experiments were approved by the Institutional Review Board of Shenzhen Institutes of Advanced Technology (registration number: SIAT-IRB-140215-H0040). All methods were carried out in accordance with guidelines of the Chinese Academy of Sciences, and all subjects submitted their informed, signed consent.

#### The natural state experiment

Thirty-four subjects (age 32.3 ± 12.3 years, height 165.9 ± 8.8 cm, 17 females) participated in the experiment. All the subjects were healthy, without any know cardiac diseases. They were asked to refrain from caffeine, alcohol, cigarettes or strenuous exercise for 2 h before the experiment. During the experiment, the subject was lying on a mattress and relaxing. Electrocardiograms (ECG), Photoplethysmograms (PPG) and beat-to-beat blood pressure were simultaneously collected from Finometer (Model II, Finapres Medical Systems B.V., the Netherlands) for 1–2 h.

#### The cold stimulation experiment

**Figure 1:**
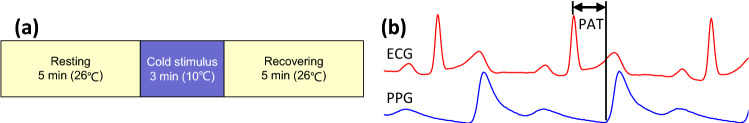
Illustration of the methods. (**a**) The procedures of the cold stimulation experiment. (**b**) Illustration of the pulse arrival time (PAT).

Fifty-five subjects (age 25.3 ± 2.2 years, height 169.0 ± 7.6 cm, and 19 females) participated in the experiment. All the subjects were healthy, without any know cardiac diseases. They were asked to refrain from caffeine, alcohol, cigarettes or strenuous exercise for 2 h before the experiment. As illustrated in Fig. 1, the subject was initially required to be lying on a mattress at normal temperature (26 °C) for 5 min, then was asked to put his/her left hand into cold water (10 °C) for 3 min, and finally to take the hand out of the water for another 5 min. During the whole 13 min, ECG, PPG and beat-to-beat blood pressure were simultaneously collected from Finometer (Model II, Finapres Medical Systems B.V., the Netherlands).

### Digital signal processing

**Figure 2:**
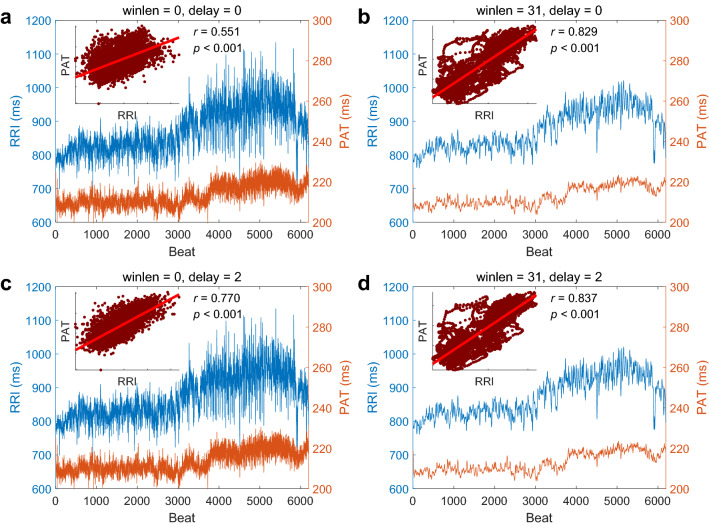
A typical example of RRI and PAT series from one subject in the natural state. winlen, the window length of the Savitzky-Golay filter (if winlen = 0, no smoothing). delay, the delayed beats of RRI series. *r,* the correlation coefficient.

All the signals were processed and analyzed offline by Matlab (The Mathworks Inc., Natick, Massachusetts, USA). The wavelet method was used to eliminate baseline drifting and interference in the raw ECG signals^16^. The successive peaks of R waves in ECG were detected by Pan-Tompkins’ method^17^ and then RRI series were obtained. For each cardiac beat, the foot of PPG was determined as the minimum point in the range from the peak of R wave in ECG to a followed distance within a third of RRI. PAT was calculated as the time interval from the peak of R wave to the foot of PPG for each cardiac cycle (Fig. 2b) by using Tang and Drinnan’s method^14,15^. Consequently, PAT and RRI were paired for each heartbeat.

In the obtained RRI series and PAT series, abnormal points caused by error detections or ectopic beats were modified to the average of its previous and next data points. Afterwards, the Savitzky-Golay filter was applied to smooth the beat-to-beat RRI and PAT series. The smoothness was controlled by the length of filter window, as the filtered signals were smoother when the window length was larger^18^. By setting different window length (i.e., different cutoff frequency, calculated by using Matlab function *sgolay* and *freqz*, see Table 1), a group of RRI and PAT series with different degrees of smoothness were obtained.

**Table 1:** The cutoff frequency of the Savitzky-Golay filter of different window length.

Window length (beat)	5	7	15	31	61	127	255	513
Cutoff frequency (beat^-1^)	0.2379	0.1600	0.0717	0.0344	0.0175	0.0084	0.0042	0.0021

### Correlation analysis

For each paired RRI and PAT series, Kolmogorov–Smirnov test was used to test the normality, and then Pearson correlation coefficient (CC) was calculated as below:

1 $$ r = \frac{{\mathop \sum \nolimits_{i = 1}^{n} \left( {x_{i} - \overline{x}} \right)\left( {y_{i} - \overline{y}} \right)}}{{\sqrt {\mathop \sum \nolimits_{i = 1}^{n} \left( {x_{i} - \overline{x}} \right)^{2} } \sqrt {\mathop \sum \nolimits_{i = 1}^{n} \left( {y_{i} - \overline{y}} \right)^{2} } }} $$

where *y* is the RRI series, *x* is the PAT series, and *n* is the length of RRI or PAT series, i.e., the number of cardiac beats.

The correlation analysis was also applied in single beat steps (1) when the RRI series were delayed several beats or/and (2) when both the RRI and PAT series were smoothed by the Savitzky-Golay filter.

### Blood pressure estimation

After obtaining the results of the correlation between PAT and RRI, we modified the conventional PAT based model (Model 1)^8^ for blood pressure estimation, to a new model with RRI (or heart rate) involved to improve the accuracy. Different from Cattivelli’s model (Model 2)^19^, the new model takes into account the phase effect that the RRI is *k* beats leading to the PAT (Model 3), where *k* = 2 or 3 which depends on the best delay for each subject. The formulas of the three models are listed below, where *a* and *b* are regression coefficients and C is a constant (i.e., the intercept).

2 $$ {\text{Model}}\;1:{\text{BP}}\left( n \right) = a \times {\text{PAT}}\left( n \right) + C $$

3 $$ {\text{Model}}\;2:{\text{BP}}\left( n \right) = a \times {\text{PAT}}\left( n \right) + b \times {\text{RRI}}\left( n \right) + C $$

4 $$ {\text{Model }}3:{\text{BP}}\left( n \right) = a \times {\text{PAT}}\left( n \right) + b \times {\text{RRI}}\left( {n - k} \right) + C, k = 2,3 $$

The data in the natural state experiment and the cold stimulation experiment were used for the three models. For each subject, the data including PAT, RRI and blood pressure were regressed by using tenfold cross validation. That is, the data were randomly divided into 10 subsets with equal size, of which 9 subsets were used for training the models and the left one was used for testing, and then this process was repeated 10 times so that each subset was tested exactly once. We then used Kolmogorov–Smirnov test to test the normality, and compared the performance of the three models by calculating their mean error (ME), standard deviation (SD) of errors, mean absolute error (MAE) and root-mean-square error (RMSE).

5 $$ {\text{ME}} = \frac{1}{n}\mathop \sum \limits_{i = 1}^{n} \left( {y_{i} - x_{i} } \right) $$

6 $$ {\text{SD}} = \sqrt {\frac{1}{n - 1}\mathop \sum \limits_{i = 1}^{n} \left( {y_{i} - x_{i} - {\text{ME}}} \right)^{2} } $$

7 $$ {\text{MAE}} = \frac{1}{n}\mathop \sum \limits_{i = 1}^{n} \left| {y_{i} - x_{i} } \right| $$

8 $$ {\text{RMSE}} = \sqrt {\frac{1}{n - 1}\mathop \sum \limits_{i = 1}^{n} \left( {y_{i} - x_{i} } \right)^{2} } $$

where *y* is the estimated blood pressure by the model, *x* is the reference value measured by the Finometer device and *n* is the number of total heartbeats for each subject.

## Results

### Correlation between PAT and delayed RRI in natural state

A typical example of beat-to-beat RRI and PAT series from one subject in the natural state experiment is shown in Fig. 2. It is obvious that the PAT changes along with the RRI beat by beat and the CC is 0.551 (Fig. 2a). When the RRI series are delayed 2 beats, the CC is 0.770, much higher than that of no delay (Fig. 2a and 2c), although 2 beats are negligible to influence the whole series which contains more than 6000 beats. This means the RRI is 2 beats advanced to the PAT, i.e., the RRI changes first and the PAT changes 2 beats later.

**Figure 3:**
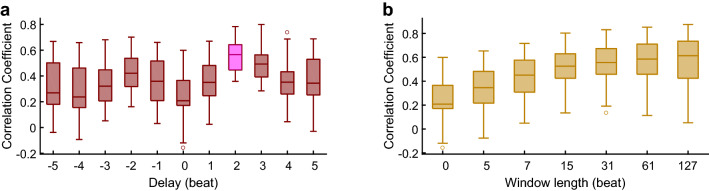
The correlation coefficients between RRI and PAT with different delays or different smoothness. (**a**) The correlation coefficients between PAT and delayed RRI for all subjects in the natural state. Delay is the delayed beats of RRI series. (**b**) The correlation coefficients between smoothed RRI and smoothed PAT for all subjects in the natural state. Window length is the window length of the Savitzky-Golay filter. If window length = 0, no smoothing.

When different delays were given to the raw RRI series for each subject, the CC between the PAT and the delayed RRI had corresponding changes. The best delay was found if the CC reached a maximum. For 28 subjects, the best delay is 2 beats; for the other 6 subjects, the best delay is 3 beats; and thus the best delay for all subjects is 2.18 ± 0.40 beats. Figure 3a shows the distribution of CC for all subjects with different delays. It is obvious that the best delay for all subjects is about 2 beats, which means the RRI is statistically about 2 beats advanced to the PAT.

### Correlation between smoothed RRI and smoothed PAT in natural state

As shown in Figs. 2a and b, when no smoothing is applied, the CC between the RRI and the PAT is 0.551, and after smoothing the RRI and the PAT series, the CC increases to 0.829 which is much higher than that of no smoothness. This is intuitive that the changes of PAT follow the changes of RRI. The smoothing operation ignores the high frequency components that changes fast and enhances the low frequency components that changes slowly. After smoothing, the general trends are clearly shown and the CC becomes higher.

When different smoothness were applied for each subject, the CC between the smoothed RRI and the smoothed PAT had corresponding changes. Figure 3b shows the distribution of CC for all subjects with different smoothness. It is obvious that when the RRI and the PAT becomes smoother, the CC goes higher. This indicates that the RRI and the PAT are more correlated in the low frequency than in the high frequency.

### Correlation between RRI and PAT with different delays and different smoothness

Considering the delays and the smoothness together, from Fig. 2, we can obtain the following facts: (1) the CC between RRI and PAT is low when no delaying or smoothing is applied; (2) the CC between RRI and PAT is higher when the RRI is delayed or both RRI and PAT are smoothed; (3) the CC between RRI and PAT doesn’t show better results when both delaying and smoothing are applied than when either delaying or smoothing is applied. We can thus infer that the delaying and the smoothing operation don’t have the superposition effect that both two is better than either one. The reason probably is that the RRI is only about 2 beats advanced to the PAT whereas these 2 beats is smoothed and neglected if the window length of the Savitzky-Golay filter is much larger than 2.

**Figure 4:**
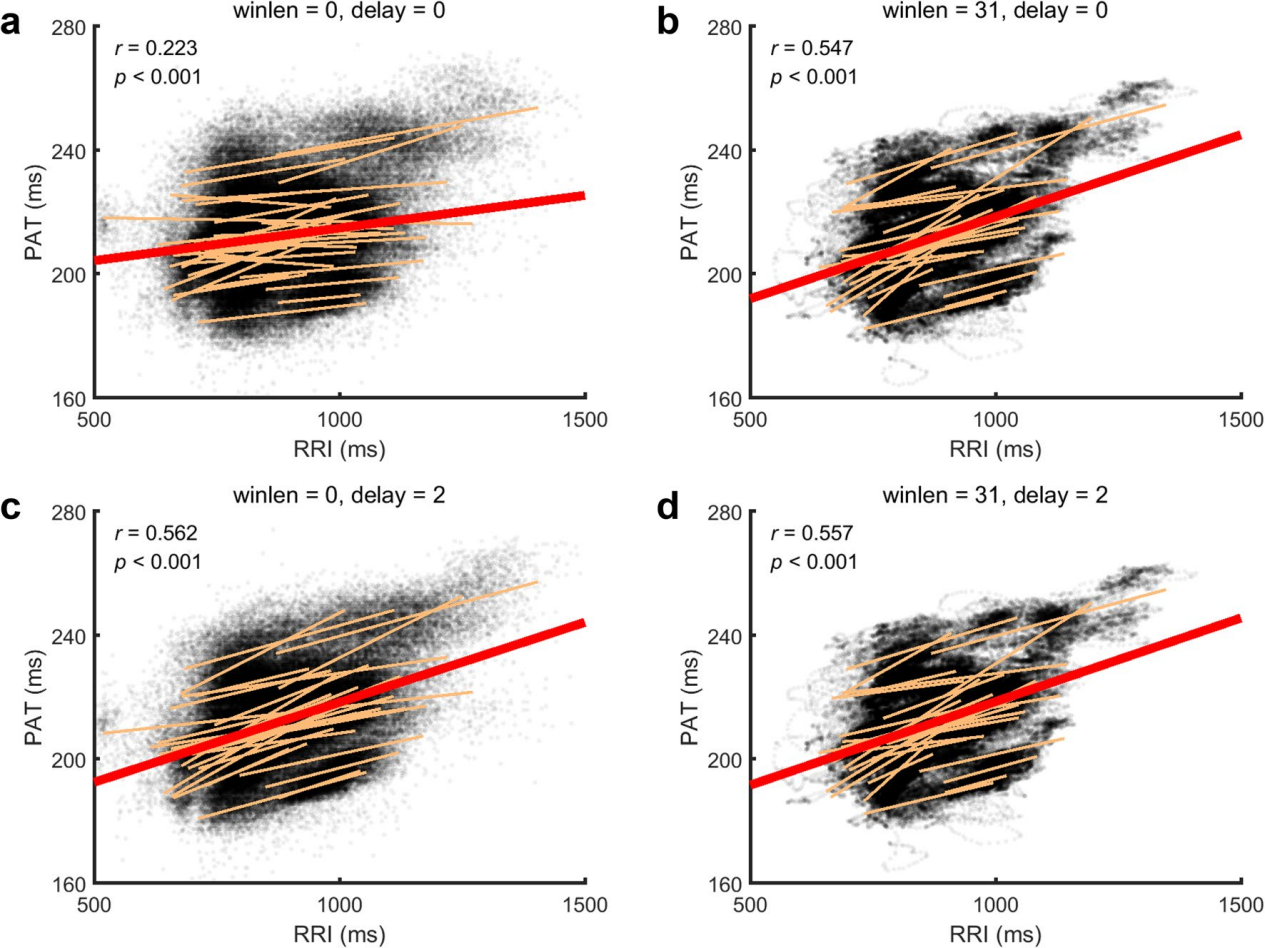
Scatter plots and linear regressions of RRI versus PAT for all subjects in the natural state. The thin yellow lines are the regression lines for individual subjects. The thick red lines are the regression lines for all subjects. winlen, the window length of the Savitzky-Golay filter (winlen = 0, no smoothing). delay, the delayed beats of RRI series. *r,* the correlation coefficient.

Not limited to the case in Fig. 2, those facts are common in all subjects. As shown in Fig. 4, the data for all subjects are pooled together. When no delaying and no smoothing, the CC between RRI and PAT is 0.223. When the RRI is delayed 2 beats, the CC between the PAT and the delayed RRI is 0.562, much higher than that of no delaying. When both RRI and PAT are smoothed, the CC between the smoothed RRI and the smoothed PAT is 0.547, much higher than that of no smoothing. When both delaying and smoothing are applied, the CC is 0.557, almost the same as that of either delaying or smoothing.

**Figure 5:**
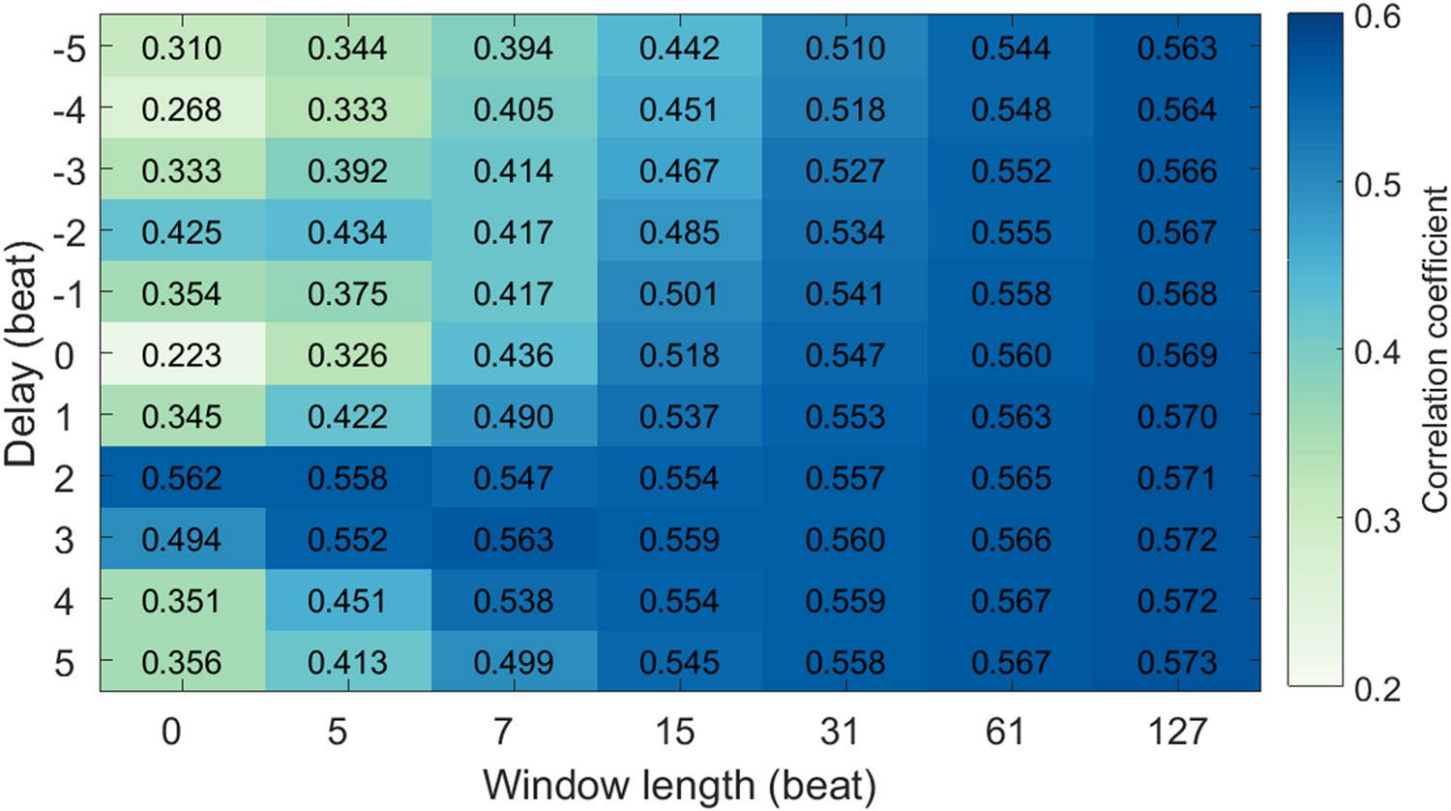
The correlation coefficients between RRI and PAT with different delays and different smoothness for all subjects in the natural state. The presented data are averaged over all subjects. Window length, the window length of the Savitzky-Golay filter (if 0, no smoothing). Delay, the delayed beats of RRI series.

We can clearly see the interaction of the delaying and the smoothing operations in Fig. 5. When no smoothing is applied, the best delay is 2 beats, clearly seen and much higher than its neighbors. As the smoothness increases, (i.e., the window length of the Savitzky-Golay filter increases), the best delay is blurred, less noticeable and not so different from its neighbors. In a word, the smoothing reduces the delaying effect. On the other hand, in general, the CC between the smoothed RRI and the smoothed PAT increases as the smoothness increases. This indicates that the RRI and the PAT are more correlated in the low frequency although they are also correlated in high frequency with a delay.

### Correlation between RRI and PAT under cold stimulation

**Figure 6:**
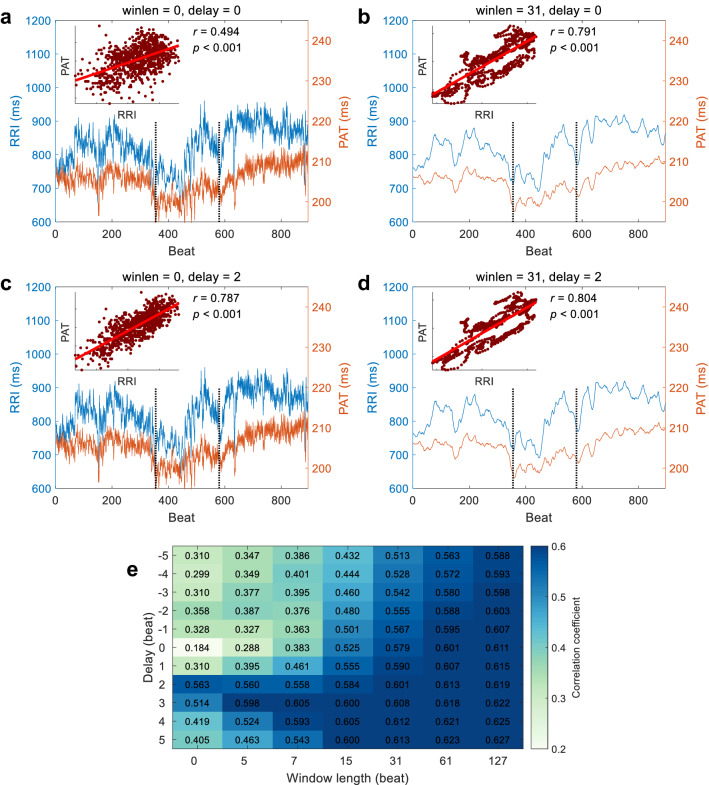
Correlation between RRI and PAT under cold stimulation. (**a**–**d**) A typical example of RRI and PAT series from one subject under the cold stimulation. The dotted vertical lines indicate the beginning and the end of the cold stimulation. winlen, the window length of the Savitzky-Golay filter (winlen = 0, no smoothing). delay, the delayed beats of RRI series. *r,* the correlation coefficient. (**e**) The correlation coefficients between RRI and PAT with different delays and different smoothness for all subjects in the cold stimulation. The presented data are averaged over all subjects. Window length, the window length of the Savitzky-Golay filter (if 0, no smoothing). delay, the delayed beats of RRI series.

Similar to the results in the natural state, the results of a typical example under cold stimulation are shown in Fig. 6. It is obvious that (1) the CC between RRI and PAT is larger when the RRI was delayed 2 beats, which implies that the RRI is advanced to the PAT, i.e., the RRI changes first and the PAT changes later (2) and the CC between RRI and PAT increases when smoothing is applied.

The interaction of the delaying and the smoothing operations under cold stimulation are also similar to that in the natural state. As shown in Fig. 6e, when no smoothing is applied, the best delay is 2 beats, clearly seen. As the smoothness increases, the best delay is blurred. This indicates the smoothing reduces the delaying effect, and the RRI and the PAT are more correlated in the low frequency although they are also correlated in the high frequency with a delay.

### Blood pressure estimation by using the relationship of RRI and PAT

**Figure 7:**
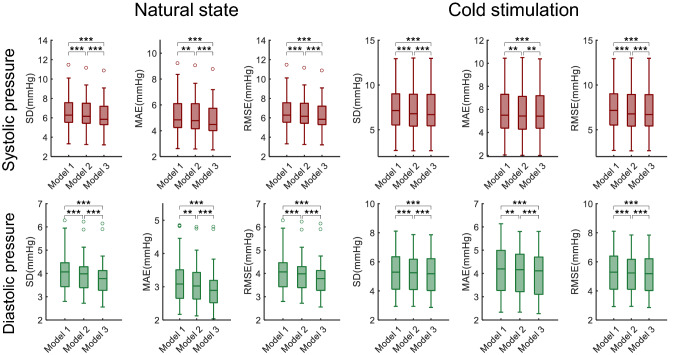
The performance of three models for blood pressure estimation (paired t-test). SD, standard deviation of errors; MAE, mean absolute error; RMSE, root-mean-square error. *, *p* < 0.05; **, *p* < 0.01; ***, *p* < 0.001.

The results of blood pressure estimation are shown in Fig. 7 and supplementary Figs. 1 and 2. It is obvious that Model 2 is better than Model 1, and Model 3 is better than Model 2. “Better” means smaller SDs (paired t-test, *p* < 0.01, the paired difference of SD for Models 2 and 3 is 0.202 ± 0.209 (mean ± SD)), smaller MAEs (paired t-test, *p* < 0.01, the paired difference of MAE for Models 2 and 3 is 0.167 ± 0.173 (mean ± SD)) and smaller RMSEs (paired t-test, *p* < 0.01, the paired difference of RMSE for Models 2 and 3 is 0.202 ± 0.210 (mean ± SD)), both for systolic pressure and diastolic pressure, both in the natural state and under cold stimulation. This indicates that the RRI (or heart rate) contributes to the blood pressure prediction, which is in line with previous studies^19–21^, and the phase lag also affects the blood pressure prediction, which is our findings mentioned above in this study.

## Discussion

In the present study, the relationships between RRI and PAT were quantitatively analyzed with different delayed beats in RRI and different signal smoothness, under the natural state and the cold stimulation. The results demonstrated that there was a strong relationship between PAT and RRI. They were greatest correlated when the RRI was delayed 2 or 3 beats; they showed better correlation in the low frequency although showed good correlation in the high frequency with a delay.

### The phase lag between PAT and RRI

The maximal correlations between the PAT and the delayed RRI occurred when the delay in RRI was 2.18 ± 0.40 beats (i.e., the best delay) for all subjects on average. This means the changes of the PAT lagged behind the RRI 2.18 beats, which is in line with the results reported by Michael J Drinnan et al*.*, who found a change in RRI followed by a corresponding change in PAT 3.17 ± 0.76 beats later during paced respiration^15^. Unlike their protocol that 15 subjects were instructed to breathe at 6 breaths per minute and ECG and PPG signals were measured only for 5 min, we analyzed the correlations based on 1–2 h of ECG and PPG recordings from 34 subjects in natural state, without any restrictions. The experimental procedures were more close to the real life situations and the results provided reliable evidences for the phase lag effect between PAT and RRI in different conditions.

The phase lag effect is especially useful for non-invasive blood pressure estimation. The commonly used model for blood pressure estimation is a linear model based on PAT^8^. Considering that the blood pressure is highly correlated with the heart rate, some researchers modified the model with heart rate involved to improve the accuracy^19–21^. However, they did not consider the phase effect and used the heart rate at the current heartbeat for blood pressure estimation. Instead, we used the RRI 2 or 3 beats preceding the current heartbeat, and obtained better results for beat-to-beat estimation of blood pressure. We think this is quite helpful in wearable medical devices for long-term monitoring.

### The frequency dependency between PAT and RRI

The correlation between the smoothed RRI and the smoothed PAT increased as the smoothness increased, which indicates that the RRI and the PAT are more correlated in the low frequency although they are also correlated in the high frequency with a delay. One possible reason is that the variations of the RRI during the natural state represent a fine tuning of the beat-to-beat control mechanisms^22^. Vagal afferent stimulation causes the reflex excitation of vagal efferent activity and inhibition of sympathetic efferent activity, and the opposite reflexes are mediated by the stimulation of sympathetic afferent activity^23^. In the HRV analysis, the vagal activity is a major contributor to the high frequency components, and the low frequency components are considered as a marker of sympathetic modulation^24^. Therefore, influence of sympathetic nervous system is one of explanations for RRI relating to PAT in the low frequency. Another possible reason is the frequency-dependent viscoelasticity of the arterial wall: decreasing heart rate (i.e., increasing the RRI) results in increasing the elasticity of the arterial wall^25^, and then increasing the PAT which indicates the elasticity of the arterial wall. These two reasons may physiologically explain the result that PAT shows higher correlations with RRI in the low frequency than in the high frequency.

### Blood pressure estimation

Blood pressure estimation is an inference of the relationship of RRI and PAT. We proposed a new model for blood pressure estimation with RRI involved and considered the phase effect (Model 3). The proposed model showed better results than the PAT-only model (Model 1) and the PAT & RRI model without phase effect (Model 2). This indicates that the heart rate (RRI) contributes to the blood pressure and has a phase leading. We think it is natural that the heart beats first and then caused the blood pressure. The results provided evidences for the common knowledge that the heart rate and the blood pressure are coupled.

A notable issue for blood pressure estimation is the difference of PTT and PAT. As aforementioned, PTT is the time that the pulse travels from one site of the body to another site. In this study PAT was measured from the R-wave of ECG to the foot of PPG, the same as reported in previous studies^20,26–28^. However, there is an argument that PTT should not be replaced by PAT for blood pressure estimation because PAT includes the pre-ejection period (PEP) which corresponds to the aortic valve opening time and consequently the PTT should be the PAT minus the PEP^29,30^. In general, whether the PEP should be included is still controversial. Although some researchers reported that PTT (PEP excluded) tracked the systolic blood pressure changes better than conventional PAT^29,30^, and excluding the PEP improved the accuracy of blood pressure by 8.4% in average^31^; some others reported that the total PAT including PEP was most suitable to determine systolic blood pressure in the normal subjects^32^, and the inclusion of PEP is necessary to facilitate accurate cuffless blood pressure prediction after exercise^33^. Regardless of these, in practice, as the PEP is difficult to measure and needs extra devices such as impedance-cardiograph (ICG), the PAT measured by ECG and PPG is still preferred and widely used for blood pressure estimation.

### Limitations

There are several limitations should be taken into account.

Firstly, the correlation study is phenomenological and the underlying mechanism is unrevealed. It is inappropriate to determine the cause and the effect of the relationship between RRI and PAT. On the other hand, the cardiovascular variables are regulated by complex physiological control systems, and some other factors such as respiratory rate^34^ was not considered in the presented study.

Secondly, the presented results are statistically significant and may not applicable to each single subject due to individual differences. For example, in Fig. 7, there are several subjects with Model 3 worse than Model 2, which is opposite to the average. This may be due to some other regulatory mechanisms involved in the regulation of RRI and PAT, such as vascular tone, peripheral resistance, blood pressure, breath rate, blood flow and hormones^35^.

## Conclusions

We investigated the correlations between the RRI and the PAT derived from ECG and PPG recordings from 34 subjects in the natural state and 55 subjects under the cold stimulation. We found a strong relationship between the RRI and the PAT, with PAT changes following RRI changes about 2.18 ± 0.40 beats delay statistically. The low frequency components of PAT and RRI showed better correlations than the high frequency components. Furthermore, when involving RRI with the phase effect, the proposed PAT based model showed better performance for blood pressure estimation. These results might help to understand the relationships associated with the regulatory mechanisms of different cardiovascular factors, and would provide useful suggestions for clinical practice, such as non-invasive cuffless blood pressure estimation.

## Supplementary Information

Supplementary Information.
