# Supplementary material for: A correlation study of beat-to-beat R-R intervals and pulse arrival time under natural state and cold stimulation
Source: Sci Rep. 2021 May 27;11:11215. doi: 10.1038/s41598-021-90056-2 (PMC8159926; doi:10.1038/s41598-021-90056-2)
Supplement: Supplementary file 1 — Supplementary Information. [file 41598_2021_90056_MOESM1_ESM.docx]

Supplementary information for

**A Correlation Study of Beat-to-beat R-R Intervals and Pulse Arrival Time under Natural State and Cold Stimulation**

Rong-Chao Peng *et al*

**Supplementary Figure 1. The performance of three models for blood pressure estimation (paired t-test).** The slope lines indicate paired data. SD, standard deviation of errors; MAE, mean absolute error; RMSE, root-mean-square error. *, *p*<0.05; **, *p*<0.01; ***, *p*<0.001.

**Supplementary Figure 2. Examples of Bland-Altman plots displaying the differences between measured and estimated systolic blood pressure.**
